# The genetic analysis of Chinese patients with clonal cytopenias using targeted next-generation sequencing

**DOI:** 10.1186/s13039-021-00572-z

**Published:** 2021-11-18

**Authors:** Lijuan Zhang, YuYe Shi, Yue Chen, Shandong Tao, Wenting Shi, Zhengmei He, Kankan Chen, Chunling Wang, Liang Yu

**Affiliations:** 1 Department of Hematology, The Affiliated Huaian No.1 People’s Hospital of Nanjing Medical University, Huai’an, 223300 Jiangsu People’s Republic of China; 2 Key Laboratory of Hematology of Nanjing Medical University, Nanjing, 210029 Jiangsu People’s Republic of China

**Keywords:** Cytopenia, Clonal hematopoiesis, Myeloid neoplasms, Next-generation sequencing

## Abstract

**Background:**

Clonal hematopoiesis (CH) can be found in various myeloid neoplasms (MN), such as myelodysplastic syndromes (MDS), myelodysplastic syndromes/myeloproliferative neoplasms (MDS/MPN), also in pre-MDS conditions.

**Methods:**

Cytogenetics is an independent prognostic factor in MDS, and fluorescence in-situ hybridization (FISH) can be used as an adjunct to karyotype analysis. In the past 5 years, only 35 of 100 newly diagnosed MDS and MDS/MPN patients were identified abnormalities, who underwent the FISH panel. In addition, we examined a cohort of 51 cytopenic patients suspected MDS or MDS/MPN with a 20-gene next generation sequencing (NGS), including 35 newly diagnosed MN patients and 16 clonal cytopenias of undetermined significance (CCUS) patients.

**Results:**

Compared with the CCUS group, the MN group had higher male ratio (22/13 vs 10/6), cytogenetics abnormalities rate (41.4% *vs* 21.4%) and frequency of a series of mutations, such as *ASXL1* (28.6% *vs* 25%), *U2AF1 (*25.7% *vs* 25%), *RUNX1* (20% *vs* 0.0%); also, higher adverse mutations proportion (75% vs 85.2%), and double or multiple mutations (54.3% vs 43.75%). There were 7 MN patients and 4 CCUS patients who experienced cardio-cerebrovascular embolism events demonstrated a significant difference between the two groups (25% *vs* 20%). Ten of the 11 patients had somatic mutations, half had DNA methylation, while the other half had RNA splicing. Additionally, six patients had disease transformation, and four patients had mutated *U2AF1*, including two CCUS cases and two MDS-EB cases. Following up to January 2021, there was no significant difference in over survival between the CCUS and MN groups.

**Conclusion:**

NGS facilitates the diagnosis of unexplained cytopenias. The monitoring and management of CCUS is necessary, also cardio-cerebrovascular embolism events in patients with CH need attention in the clinical practice.

## Introduction

Myeloid Neoplasms (MN) are a group of hematological diseases, including acute myeloid leukemia (AML), myelodysplastic syndromes (MDS), myeloproliferative neoplasms (MPN) and myelodysplastic syndromes/myeloproliferative neoplasms (MDS/MPN) [1]. The diagnosis of MDS and MDS/MPN is always challenging. MN originates from hematopoietic stem/progenitor cells (HSCs) [2]. Multi-step accumulation of genetic alterations in HSCs progressively confers growth advantages to certain clones and leads to a state of "clonal hematopoiesis (CH)" that can either exist transiently or last for many years [3]. MN usually develops via a multi-step genetic process whereby mutations mainly affecting responses to DNA damage, transcription, RNA splicing, and cytokine signalling.

Unexplained cytopenias have recently become a common and perplexing clinical problem, posing challenges in diagnosis, monitoring, and therapy. At the population level, even mild cytopenias appear to be associated with an increase in morbidity and all-cause mortality [4]. This link between blood count and morbidity and mortality has been studied most extensively in elderly people, but it also holds true for neutropenia and thrombocytopenia [5, 6]. Patients with unexplained cytopenias have stable blood counts for years and have no change in life expectancy or quality of life as a result of the cytopenias; however, some patients eventually suffer clinical consequences and progression to an overt hematologic neoplasm such as MDS or AML.

In the past decade, Next-generation sequencing (NGS) has become a useful tool in mutational analysis for patients with unexplained cytopenias, providing help to identify the etiology of unexplained cytopenias in some patients. Clonal cytopenias of undetermined significance (CCUS), also called MDS precursor states, are characterized by refractory cytopenia and CH, have been gradually concerned by clinicians.

In this study, we retrospectively analyzed 51 cases of clonal cytopenias, including 35 MN cases and 16 CCUS cases. All the patients underwent the targeted NGS panel associated with MDS mutations. The clinical characteristics and gene mutational profile were summarized. The aim of this study was to investigate the differences between CCUS and MN patients, providing data for clinical practice.

## Materials and methods

### Patients

Patients with cytopenias in the Hematology Department of the Affiliated Huaian No.1 People's Hospital of Nanjing Medical University, except non-neoplastic causes of cytopenias, including vitamin deficiency, drugs, infection, inflammation, and immunologic disorders, when the diagnosis of MDS and MDS/MPN was highly suspected, were enrolled in this study between January 2018 and January 2020. Except for the morphology (at least two puncture sites), biopsy and cytogenetics (karyotype analysis or fluorescence in-situ hybridization), the targeted NGS panel associated with MDS mutations using bone marrow (BM) samples was also recommended. The diagnostic criteria for MDS and MDS/MPN overlap disorders were according to the 2016 revision of the WHO classification [1]. The Prognostic models of MDS were according to the revised International Prognostic Scoring System (IPSS-R). Patients in very low-, low-or intermediate-risk groups are regarded as lower-risk MDS patients, and high-and very high-risk groups as higher-risk MDS. CCUS patients present with mild (below 10%) dysplasia, fewer BM blasts (below 5%), and one or more MDS-related mutations. And the diagnostic criteria were according to the literature [7].

### Targeted gene panel sequencing

Next generation sequencing of gene mutations was done with standard second-generation sequencing technology on an Illumina MiSeq System (Illumina, San Diego, CA) high-throughput sequencing platform. The 20-gene panel covers the commonly MDS-related mutations, including *ASXL1, BCOR, EZH2, TET2, KRAS, NRAS, DNMT3A, ETV6, IDH1, IDH2, RUNX1, CBL, JAK2V617F, SRSF2, SF3B1, U2AF1, ZRSR2, STAG2, p53 and SETBP1*.

### Observation indexes

A series of indexes were surveyed, including the age and sex of patients, the diagnosis, the blood cell counts, BM blasts ratio, dysplasia, ringed sideroblasts (RS), the cytogenetics, the mutation type, allele variation frequency (VAF), the treatment and the clinical outcome. In addition, the cardio-cerebrovascular embolism events that occurred in the whole disease process should be observed.

### Statistical analyses

Statistical analyses were conducted using SPSS software version 19.0 (SPSS, Inc., Chicago, USA). The association of mutations with clinical characteristics was analyzed by the χ2 test (or Fisher’s exact test for low cell counts), whereas continuous variables between groups were compared by using the t test. The over survival (OS) was calculated from the date of diagnosis to death from any cause or the last known follow-up. The Kaplan–Meier test was used for univariate survival analysis and differences were assessed by log-rank analysis. Two-tailed *P* < 0.05 was considered significant.

## Results

### Patient characteristics

A total of 35 cases of newly diagnosed MN were enrolled, 30 with MDS, and 5 with MDS/MPN. Of the MDS patients, there were 3 patients with MDS-U, 1 patient with MDS-SLD, 8 patients with MDS-MLD, 2 patients with MDS-RS, 10 patients with MDS-EB1, 6 patients with MDS-EB2. According to the IPSS-R, there were 22 out of 30 patients (73.3%) with a higher-risk prognosis. Based on the targeted gene panel sequencing, 16 CCUS cases were diagnosed. The detailed characteristics of the 16 CCUS cases and 35 MN cases are summarized in Tables 1 and 2 respectively. The MN patients had a median age of 68 years old (range 13–87) at diagnosis, while the CCUS patients were younger, with a median age of 63 years old (range 33–78). However, there were no significant differences *(P* ˃ 0.05). There were mainly male patients (62.6%), especially in MN patients. In the CCUS group there were 10 male and 6 female patients, while in the MN group there were 22 males and 13 female patients (Table 3).

**Table 1:** Characteristics of 16 CCUS cases

No	Age/sex	WBC (× 10^9^/L)	Hb (g/L)	PLT (× 10^9^/L)	BM blasts (%)	Dysplasia (%)	RS	Cytogenetics	Mutations	VAF (%)
1	33/F	1.88	89	99	< 5	< 10	N	Normal	U2AF1	40.43
2	50/M	1.49	40	14	< 5	< 10	N	Normal	ASXL1, BCOR	34.63
3	66/M	8.48	113	14	< 5	< 10	N	Normal	ASXL1, TET2	77.96
4	35/M	8.86	83	45	< 5	< 10	N	Normal	ASXL1, TET2	49.51
5	43/M	4.43	93	17	< 5	< 10	N	Normal	BCOR	51.7
6	61/F	1.79	79	88	< 5	< 10	N	Normal	TET2	10.79
7	74/M	3.3	96	70	< 5	< 10	N	20q-	TET2	23.96
8	73/F	2.35	57	291	< 5	< 10	N		TET2	5.75
9	68/F	1.2	67	25	< 5	< 10	N	Normal	TP53	32.17
10	50/F	7.55	91	25	< 5	< 10	N	20q-	U2AF1	13.86
11	77/M	4.22	96	18	< 5	< 10	N		SRSF2, TET2	40.2
12	74/M	2.32	51	73	< 5	< 10	N	20q-	SF3B1	41.2
13	48/M	1.43	53	32	< 5	< 10	N	Normal	U2AF1	43.8
14	78/M	8.06	67	23	< 5	< 10	N	Normal	ZRSR2, BCOR, ASXL1, SRSF2	94.6
15	66/F	2.08	104	188	< 5	< 10	N	Normal	BCOR, DNMT3A, IDH1	29
16	54/M	1.71	55	380	< 5	< 10	N	Normal	U2AF1, TET2	18.5

*WBC* White blood cells, *Hb* haemoglobin, *PLT* platelet, *RS* ringed sideroblasts, *VAF* Variant allele frequencies, *BM* bone marrow, *N* None

**Table 2:** Characteristics of 35 MN cases

No	Age/sex	Diagnosis type	WBC (× 10^9^ /L)	HB (g/L)	PLT (× 10^9^ /L)	BM blasts (%)	Dysplasia (%)	RS	Cytogenetics	Mutations	VAF (%)
1	76/F	MDS-U	1.2	71	15	< 5	Y	N	+ 8	*DNMT3A, ASXL1, U2AF1, STAG2, BCOR, RUNX1*	33.1
2	66/F	MDS-U	2.1	32	81	< 5	N	N	-7	*U2AF1*	28.93
3	13/M	MDS-U	1.64	53	11	< 5	N	N	Normal	*TP53*	43.22
4	81/M	MDS-SLD	5.3	37	119	< 5	Y	N	-Y	N	
5	69/M	MDS-RS	2.45	54	210	< 5	N	Y	Normal	*SF3B1*	36.45
6	87/M	MDS-RS	3.64	50	349	< 5	N	Y	Normal	*SF3B1, TET2*	44.22
7	73/M	MDS-MLD	2.4	69	4	< 5	Y	N		*TET2, TP53*	7.46
8	62/M	MDS-MLD	1.06	66	12	< 5	Y	N	Normal	*RUNX1*	37.31
9	59/F	MDS-MLD	3.2	44	4	< 5	Y	N	Normal	*N*	
10	51/M	MDS-MLD	2.39	54	262	< 5	Y	N	5q-	*U2AF1*	20.3
11	85/M	MDS-MLD	2.6	84	30	< 5	Y	N	20q-	*N*	
12	80/M	MDS-MLD	6.77	81	30	< 5	Y	N	+ 8	*TP53, ASXL1, U2AF1*	87.24
13	60/M	MDS-MLD	5.82	68	18	< 5	Y	N	Normal	*ASXL1, RUNX1, EZH2*	44.6
14	71/M	MDS-MLD	7.59	84	7	< 5	Y	N		*TET2, EZH2, ASXL1, RUNX1*	71.3
15	77/F	MDS-EB-2	2.89	106	68	16	N	N	Normal	*ASXL1, EZH2*	10.83
16	75/M	MDS-EB-2	1.52	48	38	18	N	N		*ASXL1, U2AF1*	42.15
17	69/F	MDS-EB-2	0.6	48	21	19	N	N	Normal	*IDH2, SRSF2, STAG2*	57.66
18	70/F	MDS-EB-2	2.34	52	4	18.5	N	N	5q-, 7q-, + 8	*TP53, EZH2*	68.26
19	68/M	MDS-EB-2	1.97	87	182	11	Y	N	Normal	*SRSF2, ASXL1, STAG2*	61.51
20	63/F	MDS-EB-2	3.25	84	54	14.5	N	N	Normal	*STAG2, TET2*	91.91
21	82/M	MDS-EB-1	0.85	73	56	5.5	N	N	Normal	*DNMT3A, U2AF1, RUNX1, BCOR*	61.72
22	73/M	MDS-EB-1	2.89	144	109	8	N	N	-Y	N	
23	61/F	MDS-EB-1	2.46	87	12	9	N	N	Normal	*ASXL1, U2AF1*	39.33
24	68/F	MDS-EB-1	2.66	67	81	7.5	N	N	Normal	N	
25	65/F	MDS-EB-1	1.37	62	11	7.5	N	N		*ASXL1, STAG2, RUNX1*	46.17
26	55/F	MDS-EB-1	1.55	78	140	8.5	N	N	-7	N	
27	77/M	MDS-EB-1	2.61	43	143	7	N	N	Normal	*DNMT3A, RUNX1, SF3B1*	39.25
28	51/M	MDS-EB-1	1.1	60	73	6	Y	N	Normal	*STAG2, U2AF1*	45.6
29	64/M	MDS-EB-1	1.52	62	12	8	N	N	Normal	*U2AF1*	30.6
30	67/M	MDS-EB-1	1.82	48	130	7.5	Y	N	-Y	*N*	
31	66/F	MDS/MPN	3.21	78	760	8	N	N	5q-, 11q-	*N*	
32	56/M	MDS/MPN	6.97	68	79	< 5	Y	N	Normal	*DNMT3A, ASXL1*	53.19
33	64/F	MDS/MPN	1.87	71	538	17.5	N	N	5q-, 7q-, 20q-	*TP53, TET2*	56.76
34	73/M	MDS/MPN	5.82	63	49	< 5	N	N		*TET2*	41.67
35	83/M	MDS/MPN	3.06	97	22	< 5	Y	N		*SF3B1*	10.7

*WBC* White blood cells, *Hb* haemoglobin, *PLT* platelet, *RS* ringed sideroblasts, *VAF* Variant allele frequencies, *BM* bone marrow, *N* None

**Table 3:** Comparison of characteristics between CCUS and MDS patients

Variable	CCUS (n = 16)	MN (n = 35)	P
Age (y) (Median, range)	63 (33–78)	68 (13–87)	0.058
Gender (Male/Female)	10/6	22/13	< 0.001*
WBC (× 10^9^/L) (Mean, range)	3.82 (1.2–8.86)	2.87 (0.6–7.59)	0.151
Hb (g/L) (Mean, range)	77 (40–113)	68 (32–144)	0.161
PLT (× 10^9^/L) (Mean, range)	87.63 (14–380)	108.69 (4–760)	0.666
Cytogenetic abnormalities (%)	21.4% (3/14)	41.4% (12/29)	< 0.001*
Mutation rate (%)	100 (16/16)	77.1% (27/35)	< 0.001*
VAF (%)	38.00 (5.75–94.60)	45.57 (7.46–91.91)	0.264
Mutations (≥ 2, %)	43.75% (7/16)	54.3% (19/35)	< 0.001*
Adverse mutation (%)	75% (12/16)	85.2% (23/27)	0.043*
TET2	43.75% (7/16)	17.1% (6/35)	< 0.001*
ASXL1	25% (4/16)	28.6% (10/35)	< 0.001*
U2AF1	25% (4/16)	25.7% (9/35)	< 0.001*
BCOR	25% (4/16)	5.7% (2/35)	< 0.001*
DNMT3A	6.25% (1/16)	11.4% (4/35)	< 0.001*
SF3B1	6.25% (1/16)	11.4% (4/35)	< 0.001*
TP53	6.25% (1/16)	14.3% (5/35)	< 0.001*
RUNX1	0 (0.0%)	20% (7/35)	< 0.001*
STAG2	0 (0.0%)	17.1% (6/35)	< 0.001*
EZH2	0 (0.0%)	11.4% (4/35)	< 0.001*
Cardio-cerebrovascular embolism events	25% (4/16)	20% (7/35)	< 0.001*

*WBC* White blood cells, *Hb* hemoglobin, *PLT* platelet, *VAF* allele variation

frequency

**Fig. 1:**
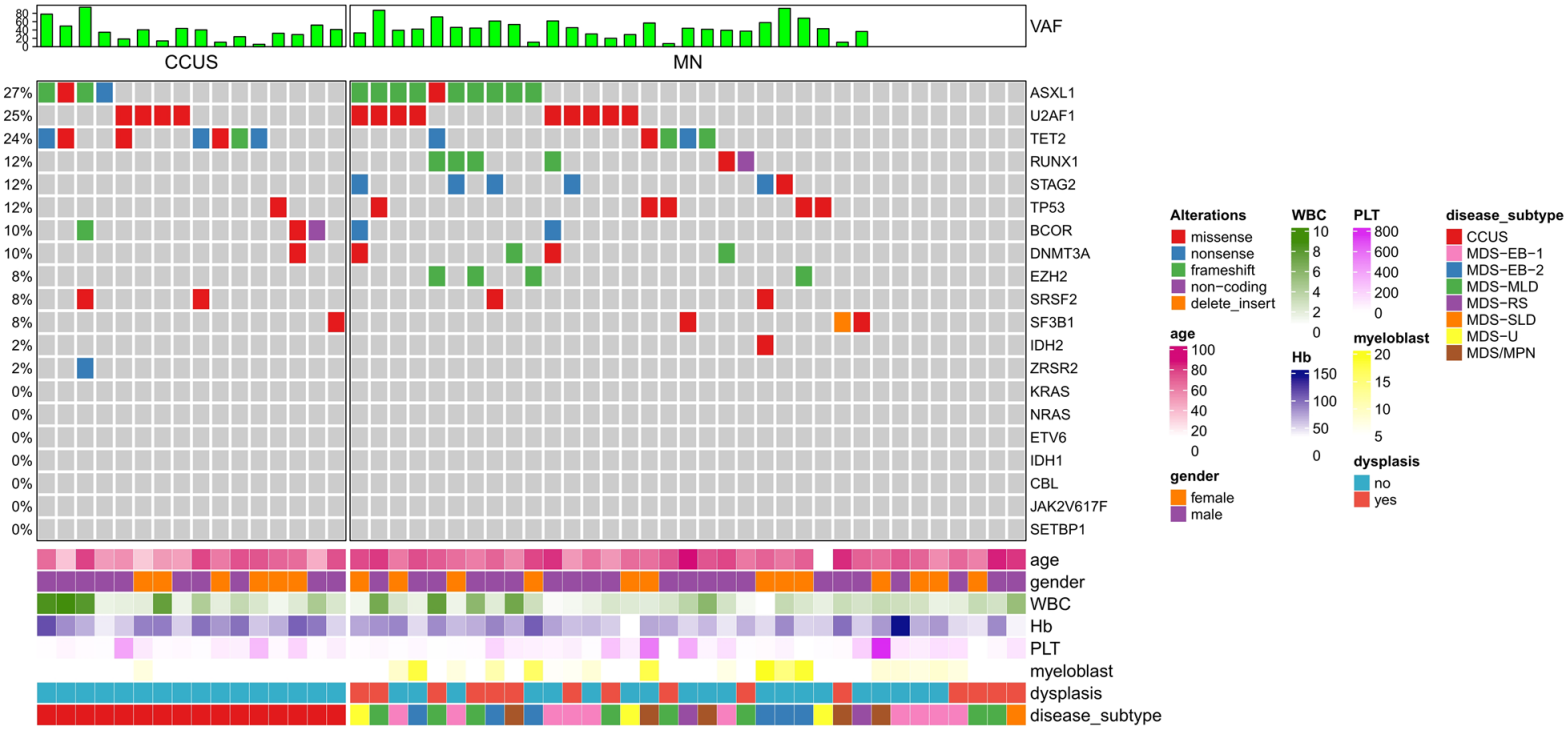
The detailed clinical characteristics and gene mutational profile of the 16 CCUS patients and 35 MN patients

All of the patients with varying degrees of cytopenias, but the blood counts (hemoglobin, white blood cell, platelet) between the CCUS and MN groups showed no significant significance (*P* ˃ 0.05). Except 2 CCUS and 6 MN cases, who failed to undergo karyotype analysis or fluorescence in-situ hybridization (FISH), cytogenetic abnormalities in the CCUS group were much lower than in the MN group (21.4% vs 41.4%, *P* < 0.001). Furthermore, all the 3 CCUS patients had 20q-. Four of 35 MDS and MDS/MPN patients (11.4%) had poor cytogenetic prognosis, such as complex karyotype and chromosome 7 abnormalities. The detailed data is presented in Table 3 and Fig. 1.

### Gene mutational profile

Twenty-seven of the 35 MN patients (77.1%) harbored at least one mutation, while all the 16 CCUS patients had positive mutations (100%), showing a significant difference between the two groups (*P* < 0.001) (Table 3), and the mutation subtypes have been presented in Fig. 1. However, the rate of double and multiple mutations in the MN group was much higher than in the CCUS group (54.3% vs 43.75%, *P* < 0.001) (Table 3). Twenty-three of the 27 mutated MN patients (85.2%) harbored at least one MDS-related adverse mutation, such as *ASXL1, U2AF1, DNMT3A, p53, EZH2, BCOR*. etc. However, 12 patients in the CCUS group had the above adverse mutations, showing a lower proportion than the MN group (75% *vs* 85.2%, *P* = 0.043) (Table 3). The MN group had a mean VAF of 45.57% (7.46%-91.91%), while the CCUS group had a mean VAF of 38.0% (5.75%-94.60%), showing no significant significance between the two groups (*P* = 0.264) (Table 3).

The mutation rates of many genes in the MN group were higher than those in the CCUS group, such as *ASXL1* (28.6% *vs* 25%), *U2AF1* (25.7% *vs* 25%), *RUNX1* (20% *vs* 0.0%), *STAG2* (17.1% *vs* 0.0%), *p*53 (14.3% *vs* 6.25%), *DNMT3A* (11.4% *vs* 6.25%), *SF3B1* (11.4% *vs* 6.25%) and *EZH2* (11.4% *vs* 0.0%), with significant differences (*P* < 0.05) (Table 3). However, compared with the MN group, the CCUS group had higher mutation rates of *TET2* (43.75% *vs* 17.1%) and *BCOR (*25% *vs* 5.7%), with significant differences (*P* < 0.05) (Table 3).

**Fig. 2:**
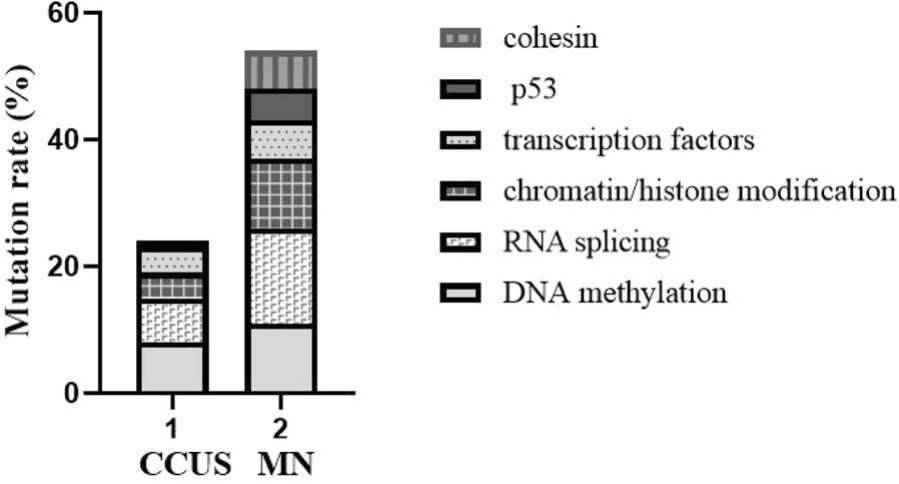
The different distribution of patients with mutated genes between CCUS group and MN group. The MN group with higher mutation rates of RNA splicing (15/27, 55.6%), DNA methylation (11/27, 40.7%), chromatin/histone modification (11/27, 40.7%), while the CCUS group with higher mutation ratios of DNA methylation (8/16, 50%) and RNA splicing (7/16, 43.75%)

According to the mutation functions and the mutation rates of the 51 patients, there were mainly six types, including DNA methylation (*TET2, DNMT3A*, and *IDH1/IDH2*), RNA splicing (*U2AF1*, *SF3B1*, *SRSF2* and *ZRSR2*), chromatin/histone modification (*EZH2* and *ASXL1*), transcription factors (*RUNX1* and *BOCR*), cohesin (*STAG2*) and tumor suppressor (*p53*). Consequently, we summarized the distribution of patients with mutated genes between the CCUS group (n = 16) and the MN group (n = 27). The results demonstrated that there was a difference in mutation distribution, with the MN group having higher mutation rates of RNA splicing (15/27, 55.6%), DNA methylation (11/27, 40.7%), chromatin/histone modification (11/27, 40.7%), and the CCUS group had higher mutation ratios of DNA methylation (8/16, 50%) and RNA splicing (7/16, 43.75%) (Fig. 2).

### Cardio-cerebrovascular embolism events

In the whole disease process, 11 of 51 patients (21.6%) experienced cardio-cerebrovascular embolism events, 6 with coronary atherosclerotic heart disease, 4 with cerebral infarction, and 1with both the complications. There were 7 MN patients and 4 CCUS patients respectively, showing significant significance between the two groups (25% vs 20%,* P* < 0.05) (Table 3). Ten of the 11 patients (90.9%) had somatic mutations, and one half of patients with DNA methylation, while the other half with RNA splicing.

### Therapy and follow-up

All of the 16 CCUS cases underwent the basic supportive treatment, such as oral cytomegalic drugs, androgen, erythropoietin and blood transfusion. Except 1 patient who was treated outside, without detailed information, 20 of 34 MN patients selected the basic supportive treatment, 13 demethylation therapy, and 1 allogeneic hematopoietic stem cell transplantation. Only 1 patient was terminated demethylation therapy due to serious infection complications, 1 died of intracranial hemorrhage during chemotherapy, and the other patients were completely relieved after 1–4 courses of chemotherapy.

**Fig. 3:**
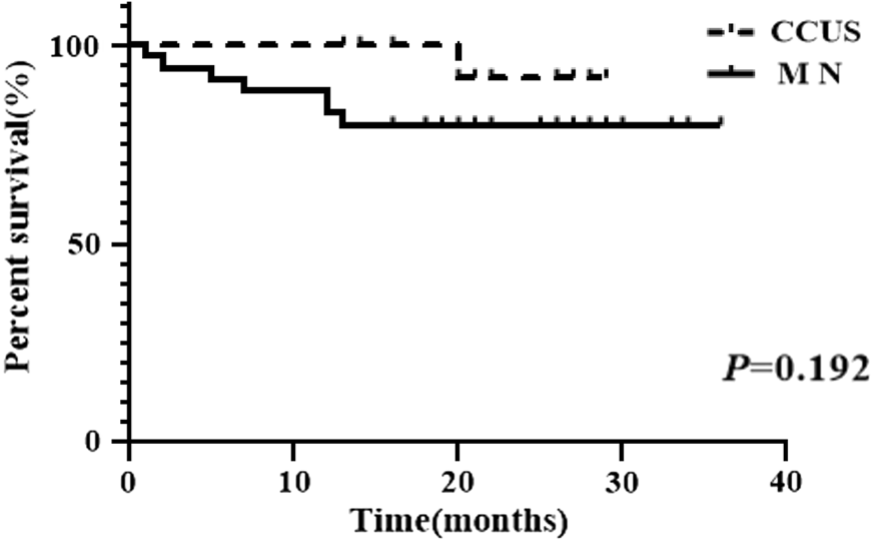
The survival rate of CCUS group and MN group. Up to January 2021, the survival rate of the CCUS group was 93.8% (15/16), while the MN group was having 80.0% (28/35). However, the OS between the CCUS group and MN group showed no significant significance (*P* = 0.192)

Up to January 2021, the CCUS group had a survival rate of 93.8% (15/16), while the MN group had an 80.0% (28/35). Because of the short follow-up period, there was no significant difference in OS between the CCUS and MN groups (Fig. 3). Two patients (12.5%) in the CCUS group had disease progression. One patient converted to MDS-EB1 and underwent allogeneic hematopoietic stem cell transplantation in another hospital, without providing detailed information. The other CCUS patient developed AML, refused chemotherapy, and died three months later (Table 4). Four patients (11.4%) in the MN group had disease progression, and all four were converted to AML (Table 4). One patient was still alive after receiving azacitidine and venetoclax treatment, while the other three patients died after receiving standard chemotherapy or basic supportive care. Furthermore, *U2AF1* mutation was found in 4 of the 6 patients (66.6%) who progressed, including 2 CCUS cases and 2 MDS-EB cases (Table 4).

**Table 4:** Characteristics of 6 cases with disease transformation

No	Age/Sex	Diagnosis	Transformation	Mutation type	Time (months)	Outcome
1	33/F	CCUS	MDS-EB1	*U2AF1*	1	Alive
2	54/M	CCUS	AML	*U2AF1*	17	Death
3	64/M	MDS-EB1	AML	*U2AF1*	2	Death
4	61/F	MDS-EB1	AML	*U2AF1*	12	Alive
5	62/M	MDS-MLD	AML	*RUNX1*	10	Death
6	56/M	MDS/MPN	AML	*DNMT3A, ASXL1*	17	Death

*CCUS* clonal cytopenias of undetermined significance, *MDS* myelodysplastic syndromes, *MDS/MPN* myelodysplastic syndromes/myeloproliferative neoplasms, *AML* acute myeloid leukemia

## Discussion

Cytopenia is very common in clinical practice. Despite a careful and organized assessment of common etiologies for blood cytopenias, such as nutritional deficiency, hematologic neoplasia, drugs, or an autoimmune disorder, there remain many unexplained cytopenias. MN is a group of hematological malignancies characterized by CH, usually with varying cytopenias. The advent of NGS can help bring clarity to the etiology of unexplained cytopenias in some patients with NGS. It has the potential to increase the detection sensitivity of small clonal populations by identifying low-frequency somatic mutations associated with malignant clones [8]. Recently, mutational analysis for patients with unexplained cytopenias is becoming common practice [9]. It was reported that 40–50 MDS-related genes have been found in the vast majority of known pathogenetic variants [10]. NGS using panels of 20 or more MDS-associated genes is now routine in clinical practice when evaluating patients with unexplained cytopenias, at least in the USA, and the detection of mutation influences hematopathologists’ interpretation of morphology [11, 12]. In this study, due to the limited economic conditions, the panel consisted of 20 MDS-related genes, mainly applied to unexplained cytopenias suspected of MDS or MDS/MPN. Thanks to the targeted NGS, a total of 30 MDS, 5 MDS/MPN and 16 CCUS cases were diagnosed combined with other laboratory examinations.

The diagnosis and prognosis of MDS mainly depend on morphology and cytogenetics. However, recurrent chromosomal abnormalities are only detected in about 50% of patients with MDS, limiting the utility of cytogenetics [13, 14]. Haferlach et al. [10] summarized a total of 944 MDS cases, and found that 80% -90% patients harbored at least one mutation, and the most common mutations were TET2, SF3B1, ASXL1, etc. Zheng et al. [15] reported that 64% (158/247) and 71% (157/221) of the mutations were detected by the 640-gene NGS panel in low-grade and high-grade MDS patients, respectively. Regarding the cytogenetics, FISH can provide additional information in cases with karyotype analysis failure, and the general panel testing includes -5/-5q, -7/-7q, + 8, 20q- and -Y. In this study, a total of 100 newly diagnosed MDS and MDS/MPN underwent the FISH panel in the past 5 years, only 35 cases (35/100) detected abnormalities. Because FISH probes are restricted to the detection of only specific abnormalities, other genetic alterations could be missed. However, the mutation rate of 35 MDS and MDS/MPN patients was up to 77.1%, with high rates of ASXL1 (28.6%), U2AF1 (25.7%), RUNX1 (20%) and TET2 (17.1%), while the rate of cytogenetic abnormalities was only 41.4%, similar to previous reports. Because of the small number, the mutation rates were not analyzed for contrast between the higher-risk and lower-risk MDS.

Gangat et al. [16] analyzed 300 MDS cases who underwent targeted sequencing, and found that 220 (73%) patients harbored one or more adverse mutations; 135 (45%) patients harbored one, 70 (23%) harbored two and 15 (5%) harbored one adverse mutation. In the study, 23 of 27 MN patients harbored one or more adverse mutations, much higher than the patients in the CCUS group (85.2% *vs* 75%, *P* = 0.043).

It has been verified that alteration of multiple genes is involved in the molecular pathogenesis of tumor development. In MN, epigenetic regulators (*DNMT3A, TET2,* and *ASXL1*, for example) are frequently mutated early, followed by alterations in gene expression (splicing factors, cohesion, and transcription regulators) and/or activation of growth signals [3]. In this study, DNA methylation mutations were the most common (8/16, 50%) in CCUS group, while RNA splicing mutations were the most common (15/27, 55.6%) in MN group (Table 3, Fig. 2). Based on a 112-gene sequencing for 511MDS patients, Li et al. [17] found that 138 (27%) patients had two mutated genes, 80 (16%) with three mutated genes and 73 (14%) with more than three mutated genes. In this study, compared with the CCUS group, the rate of double or multiple mutations was much higher in the MN group (43.75% *vs* 54.3%, *P* < 0.001) (Table 3). These data further illustrate the complex development of MN.

In overt MDS, the mutation VAF is usually higher than that found in clonal pre-MDS conditions. It was reported that the mean VAF of CCUS patients was less than 11.2% (n = 52), while approximately 30% to 40% in MDS patients (n = 115) based on a 640-gene panel [15]. In this study, the mean VAF of patients in the MN group was higher than in the CCUS group (45.57% *vs* 38.00%), but there was no significant difference (*P*˃0.05). Malcovati et al. [18] suggested that patients with CCUS had a considerably increased risk of progression to MDS or AML within 10 years. In our study, 2 of 16 CCUS patients (12.5%) suffered with disease progression in several months. One patient converted to MDS-EB1, and the other converted to AML. In addition, 1MDS/MPN and 3 MDS patients converted to AML (Table 4). Recent studies have reported that *U2AF1* mutation is related to higher rates of AML transformation and short overall survival, usually indicating poor prognosis for MDS patients [19, 20]. Coincidentally, 4 of the 6 patients (66.6%) in our study had *U2AF1* mutation, including 2 CCUS cases and 2 MDS-EB cases (Table 4). Thus, the *U2AF1* mutation should be noted not only in MDS patients, but also in CCUS patients in the follow-up.

CH is a precursor state to MN. It was reported that clonal hematopoiesis increased an individual’s risk of experiencing a myocardial infarction or stroke and of dying from a cardiovascular event, and almost doubling in the relative risk of cardiovascular disease in comparison with those without these mutant clones [21, 22]. Regarding the mechanism, it has been reported that the patients with clonal hematopoiesis, especially with DNA or histone methylation mutations, had elevated expression of a set of proinflammatory genes, including cytokines such as interleukins 6 and 1, mediators strongly implicated in promoting human cardiovascular events [23]. In our study, a total of 11 patients suffered cardio-cerebrovascular embolism events during the whole disease process, 6 with coronary atherosclerotic heart disease, 4 with cerebral infarction, and 1 with both the complications. Ten of the 11 patients (90.9%) had somatic mutations, and one half of the patients had DNA methylation, the other half had RNA splicing. In addition, we found that the rate of the MN group was higher than the CCUS group, showing significant significance (25% *vs* 20%, *P* < 0.05) (Table 3).

## Conclusion

We summarized the characteristics of 51 cytopenia cases associated with CH and discovered some differences between CCUS and MN patients, particularly in terms of gene mutational profile and rate of cardio-cerebrovascular embolism events. Genetic analysis with targeted NGS panels is extremely useful for diagnosing cytopenias that are unexplained. If sufficient funding is made available, NGS panels can be widely used in routine clinical testing, such as monitoring pre-MDS disorders or diagnosing, prognosing, and managing MN.

## Data Availability

The raw data required to reproduce these findings cannot be shared at this time as the data also forms part of an ongoing study.
